# Gamma Irradiation Increases the Antioxidant Properties of Tualang Honey Stored Under Different Conditions

**DOI:** 10.3390/molecules17010674

**Published:** 2012-01-11

**Authors:** Md. Ibrahim Khalil, Siti Amrah Sulaiman, Nadia Alam, Mohammed Moniruzzaman, Saringat Bai’e, Che Nin Man, Syed Mohsin Sahil Jamalullail, Siew Hua Gan

**Affiliations:** 1 Department of Pharmacology, School of Medical Sciences, Universiti Sains Malaysia, 16150 Kubang Kerian, Kelantan, Malaysia; Email: rasmo04@yahoo.com (M.M.); 2 Department of Biochemistry and Molecular Biology, Jahangirnagar University, Dhaka-1342, Bangladesh; Email: mibrahim12@yahoo.com (M.I.K.); 3 Department of Botany, Rajshahi University, Rajshahi-6205, Bangladesh; Email: najruc@yahoo.com; 4 School of Pharmaceutical Sciences, Universiti Sains Malaysia, 11800 Penang, Malaysia; Email: saringat@usm.my; 5 National Poison Center, Universiti Sains Malaysia, 11800 Penang; Malaysia; Email: chenin@usm.my; 6 Research Dean, Biomedical & Health Sciences Platform, Health Campus, Universiti Sains Malaysia, 16150 Kubang Kerian, Kelantan, Malaysia; Email: smohsin@kb.usm.my; 7 Human Genome Centre, School of Medical Sciences, Universiti Sains Malaysia, 16150 Kubang Kerian, Kelantan, Malaysia; Email: shgan@kck.usm.my

**Keywords:** Tualang honey, gamma irradiation, storage effects, polyphenols, radical-scavenging activities

## Abstract

This study was conducted to evaluate the effects of evaporation, gamma irradiation and temperature on the total polyphenols, flavonoids and 1,1-diphenyl-2-picrylhydrazyl (DPPH) radical-scavenging activities of Tualang honey samples (n = 14) following storage over three, six or twelve months. The mean polyphenol concentrations of the six gamma irradiated honey samples at three, six and twelve months, respectively, were 96.13%, 98.01% and 102.03% higher than the corresponding values of the eight non-gamma irradiated samples. Similarly, the mean values for flavonoids at three, six and twelve months were 111.52%, 114.81% and 110.04% higher, respectively, for the gamma irradiated samples. The mean values for DPPH radical-scavenging activities at three, six and twelve months were also 67.09%, 65.26% and 44.65% higher, respectively, for the gamma irradiated samples. These data indicate that all gamma irradiated honey samples had higher antioxidant potential following gamma irradiation, while evaporation and temperature had minor effects on antioxidant potential.

## 1. Introduction

Honey is a food product generated by honey bees (*Apis mellifera*) using nectar that is collected by the bees from various plants. For centuries, honey has been used for nutrition in different cultures and it has also been used as a traditional medicine due to its healing properties. Recently, honey has been scientifically tested and confirmed to possess functional and biological properties such as antioxidant, anti-inflammatory, antibacterial, antiviral, antiulcerative activities, antilipid and anticancer properties [1,2,3,4,5,6]. These properties are mainly attributed to phenolic constituents such as flavonoids that have antioxidant properties and radical-scavenging activities. These flavonoids are observed in all types of honeys in different proportions depending on geography, food source for the honeybee and climate [7,8,9].

Honey polyphenols such as flavonoids and phenolic acids act as free radical scavengers, peroxy radical scavengers and metal chelators [10,11]. The antioxidant activity of a number of honey samples has been reported, and it is significantly correlated with phenolic content [2]. The antioxidants reported to be present in honey include both enzymatic and non-enzymatic substances, in addition to more than 150 polyphenolic compounds including flavonoids, flavonols, phenolic acids, catechins and cinnamic acid derivatives. It also contains small amounts of other constituents such as minerals, proteins, vitamins, organic acids, and other phytochemicals which contribute to its antioxidant effects. The components in honey that are responsible for the antioxidant effects are flavonoids, phenolic acids, ascorbic acid, catalase, peroxidase, carotenoids, and products of Maillard reactions [2,12,13,14]. The quantity of these components varies widely depending on the floral and geographical origin of the honey. In addition, processing, handling, and storage of honey may influence its composition [2,15,16,17].

Malaysian Tualang honey is collected from the honey combs of Asian rock bees (*Apis dorsata*) that build their hives high up in the Tualang tree (*Koompassia excelsa*). Tualang honey is commonly used as a medicinal product [18] and food in Malaysia. Recently, the antibacterial properties of this honey have been studied and compared with other honey types [19,20]. Tualang honey has good color intensity and contains phenolic compounds that possess relatively effective antioxidant activity [21].

Many previous studies on the antioxidant activity of honey focused primarily on processed honeys. Honey flavor and color is reported to be best immediately following extraction [22]. “Raw” honey contains extraneous matter including pollen, wax and variable levels of sugar-tolerant yeasts and may contain crystals of dextrose hydrate. These substances are removed from honey in order to improve its performance in large-scale markets. Honey is prone to fermentation unless the moisture content is maintained below 17% [22], and most honey will crystallize over time unless action is taken to prevent crystallization. Granulated honey is more likely to ferment than liquid honey [23]. Raw honey, without processing, is unsuitable for large-scale marketing. Commercial honey processing includes controlled heating to destroy yeast and to dissolve dextrose crystals, together with fine straining or pressure filtration. Because the effect of heat is additive with time, the effects of processing and storage conditions on honey are often considered together [24,25,26].

Currently, the impact of processing and storage on polyphenol content and free radical-scavenging activity of honey is not well understood. The objective of this study was to determine the impact of processing, specifically evaporation and gamma irradiation, and different storage conditions on polyphenol and flavonoid content and free radical-scavenging activities of Tualang honey.

## 2. Results

### 2.1. Polyphenol Content

**Figure 1:**
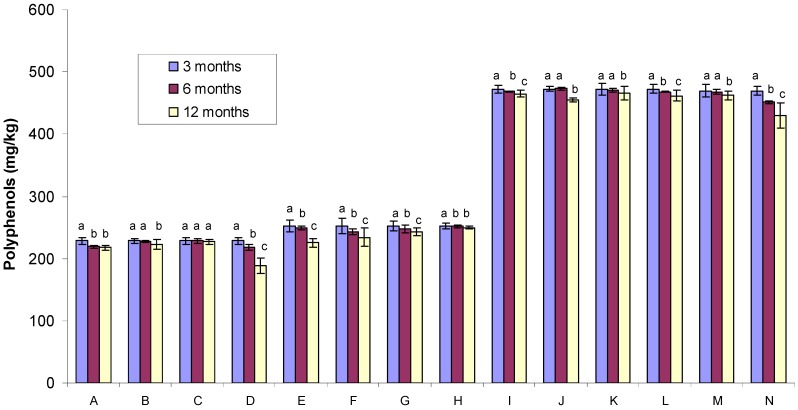
Comparison of the polyphenol content of Tualang honey samples exposed to different storage conditions (the data obtained after 6 and 12 months were compared with the base line data obtained after three months). Different letters (a, b and c) indicate significant differences (*p* < 0.05). The letters “A” to “N” indicate the sample code (Table 1).

Figure 1 compares the total phenolic (TP) content in Tualang honey samples exposed to different storage conditions and treatments. Details of the treatment conditions of Tualang honey samples were shown in Table 1. The TP content of the Tualang honey samples measured at three months (228.37–472.52 mg GAE/kg) was almost identical to that of the six month samples (218.40–473.06 mg GAE/kg). By 12 months, however, the levels tended to be lower (188.62–465.96 mg GAE/kg). Overall, all honey samples exposed to gamma irradiation tended to show higher TP content at all time points when compared to the non-gamma irradiated samples.

**Table 1:** Storage conditions (gamma irradiation, evaporation, container and temperature) for each honey sample.

Sample no.	Gamma irradiated condition	Glass container	Temperature *	Sample code
1	Non-evaporated and non-gamma irradiated	Dark bottle	Cold room	A
2	Non-evaporated and non-gamma irradiated	Dark bottle	Room temperature	B
3	Non-evaporated and non-gamma irradiated	Clear bottle	Room temperature	C
4	Non-evaporated and non-gamma irradiated	Clear bottle	Cold room	D
5	Evaporated and non-gamma irradiated	Dark bottle	Cold room	E
6	Evaporated and non-gamma irradiated	Clear bottle	Cold room	F
7	Evaporated and non-gamma irradiated	Clear bottle	Room temperature	G
8	Evaporated and non-gamma irradiated	Dark bottle	Room temperature	H
9	Evaporated and gamma irradiated	Dark bottle	Room temperature	I
10	Evaporated and gamma irradiated	Clear bottle	Room temperature	J
11	Evaporated and gamma irradiated	Dark bottle	Cold room	K
12	Evaporated and gamma irradiated	Clear bottle	Cold room	L
13	Sachet (evaporated and gamma irradiated)	Sachet	Room temperature	M
14	Sachet (evaporated and gamma irradiated)	Sachet	Cold room	N

* Cold room: 4–8 °C; room temperature: 25–30 °C.

### 2.2. Flavonoid Content

**Figure 2:**
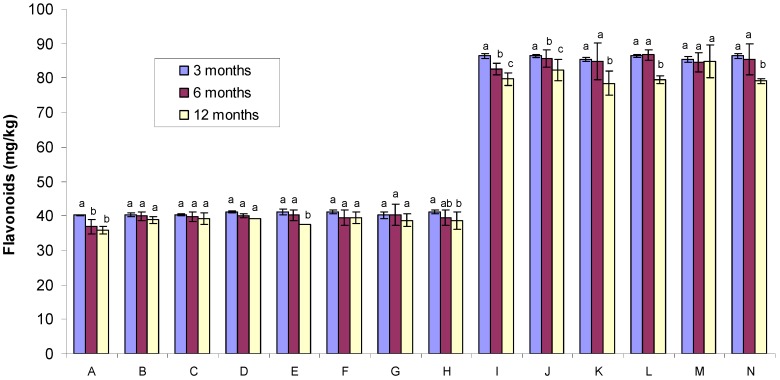
Comparison of the flavonoid content of Tualang honey samples exposed to different storage conditions (the data obtained after 6 and 12 months were compared with the baseline data obtained after three months). Different letters (a, b and c) indicate significant differences (*p* < 0.05). The letters “A” to “N” indicate the sample code (Table 1).

The flavonoid content of Tualang honey at three months ranged from 40.23 to 86.42 mg GAE/kg and tended to decrease by six months (from 36.88 to 86.71 mg GAE/kg) and twelve months (from 35.92 to 84.87 mg GAE/kg) (Figure 2). Flavonoid content tended to decrease over time for the following conditions of storage: (1) non-evaporated and non-gamma irradiated samples stored in dark bottles and in the cold room (sample code A); (2) evaporated and non-gamma irradiated samples stored in dark bottles and in the cold room (sample code E); (3) evaporated and non-gamma irradiated samples stored in dark bottles and at room temperature (sample code H); (4) evaporated and gamma irradiated samples stored in dark bottles and at room temperature (sample code I); (5) evaporated and gamma irradiated samples stored in clear bottles and at room temperature (sample code J); (6) evaporated and gamma irradiated samples stored in dark bottles and in the cold room (sample code K); (7) evaporated and gamma irradiated samples stored in clear bottles and in the cold room (sample code L) and (8) evaporated and non-gamma irradiated sachet samples stored in the cold room (sample code N). For the rest of the samples, there was no significant difference in the flavonoid content over time. These data indicate that storage conditions and containers play a vital role in maintaining the flavonoid content in honey samples.

### 2.3. DPPH Free Radical-Scavenging Activity of Gamma Irradiated Honeys

**Figure 3:**
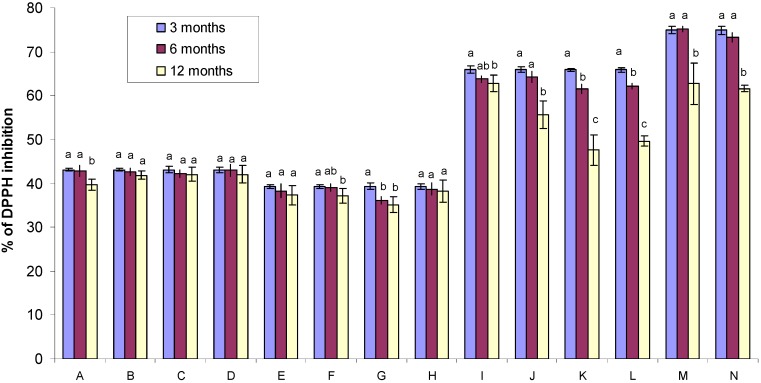
Comparison of the DPPH radical-scavenging activities of Tualang honey samples exposed to different storage conditions (data obtained after 6 and 12 months were compared with the base line data obtained after three months). Different letters (a, b and c) indicate significant differences (*p* < 0.05). The letters “A” to “N” indicate the sample code (Table 1).

All Tualang honey samples have high antioxidant activities when measured using DPPH free radical-scavenging assays. The percentage of DPPH radical-scavenging activity at three months, six months and twelve months was 39.30–74.89%, 36.09–75.13% and 35.12–62.81%, respectively (Figure 3).

**Figure 4:**
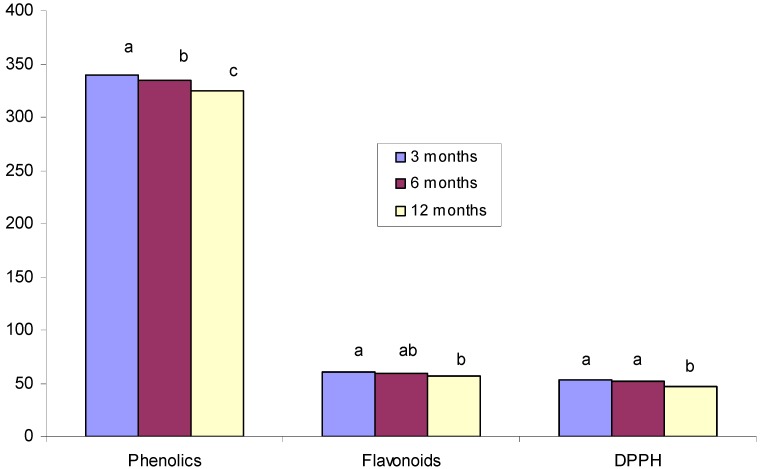
Mean phenolic and flavonoid contents and DPPH free radical-scavenging activities in Tualang honey samples (n=14) stored in different conditions for three, six or twelve months. Different letters (a, b and c) indicate significant differences (*p* < 0.05).

Figure 4 summarizes the mean levels of polyphenols, flavonoids and DPPH radical-scavenging activities of the honey samples (n = 14) when exposed to different storage conditions. In general, all measurements were highest at three months and tended to decrease by twelve months. For example, the mean polyphenol level was highest (339.48 mg/kg) when measured at three months, and it decreased to 324.98 mg/kg by twelve months. For flavonoids, the mean value at three months was 60.15 mg/kg, and this value was at its lowest (56 mg/kg) by twelve months. For DPPH, the highest radical-scavenging activities were also observed at three months (53.06%) while the lowest value, 46.68%, was observed at twelve months. These data indicate that the antioxidant properties of honey tend to decrease when honey samples are stored over a long period of time.

In the case of DPPH radical-scavenging activities, no significant decrease was found over three, six or twelve months in the following conditions: (1) non-evaporated and non-gamma irradiated samples stored in dark bottles and at room temperature (sample code B); (2) non-evaporated and non-gamma irradiated samples stored in clear bottles and at room temperature (sample code C); (3) non-evaporated and non-gamma irradiated samples stored in clear bottles and in the cold room (sample code D); (4) evaporated and non-gamma irradiated samples stored in dark bottles and in the cold room (sample code E) and (5) evaporated and non-gamma irradiated samples stored in dark bottles and at room temperature (sample code H). The rest of the samples showed a significant decrease in DPPH radical-scavenging activities over time. These data indicated that storage conditions and containers play a vital role in maintaining the DPPH radical scavenging-activities of honey samples.

Overall, storage temperature and containers (dark or clear bottles) did not have significant effects when compared to gamma irradiation on the TP and flavonoid content and DPPH radical-scavenging activities. For honey samples packed in sachets, the phenol and flavonoid content as well as the DPPH radical-scavenging activities appeared high, indicating that honey processed into sachet forms maintained their antioxidant properties. However, our findings also showed that it is advisable to store the sachets at 4–8 °C to maintain the antioxidant activities.

By six months, the mean content of the polyphenols in the 14 honey samples significantly decreased by 1.41% followed by a further significant reduction (4.27%) at twelve months (Figure 4). For flavonoids, a significant decrease of 1.84% and 5.98% was observed after six and twelve months respectively. For DPPH radical-scavenging activities, the levels decreased by 2.69% at six months, followed by a much higher reduction (12.03%) at twelve months. These data again indicate that the antioxidant properties of honey decreases when stored over a long period of time.

All gamma irradiated Tualang honey samples showed higher DPPH free radical-scavenging activities when compared to the non-gamma irradiated samples (Figure 3). The percentage of DPPH radical-scavenging activities of non-gamma irradiated Tualang honey samples at three, six and twelve months was 39.3–43.1%, 36.1–43.0% and 35.1–42.1% when compared to gamma irradiated samples which had higher values (at 65.8–74.9%, 61.5–75.1% and 47.6–62.8% respectively). Pearson’s correlation matrix showed a highly significant correlation between the TP content, flavonoid content and DPPH radical-scavenging activities (r = 0.98, *p* = 0.01) (Table 2).

**Table 2:** Correlation matrix among the mean polyphenol and flavonoid contents and DPPH radical-scavenging activities (n=3, three months, six months or twelve months) of the 14 investigated honey samples.

	Polyphenols	Flavonoids	DPPH
Polyphenols	1	0.98(**)	0.98(**)
Flavonoids	0.98(**)	1	0.98(**)
DPPH	0.98(**)	0.98(**)	1

** Correlation is significant at the 0.01 level (2-tailed).

## 3. Discussion

Tualang honey has high levels of TP and flavonoids as well as DPPH free radical-scavenging activities. However, a significant variation in these antioxidant activities was observed when the samples were exposed to different storage conditions.

The highest TP content was observed in evaporated and gamma irradiated Tualang honey samples (472.52 mg GAE/kg). This content is lower than that reported by Kishore *et al.* [27] (839.60 mg GAE/kg) but higher than that reported by Mohamed *et al.* [28] (251.70 mg GAE/kg) for similarly treated Tualang honey. These variations may be due to the different regions where and seasons when the Tualang honey samples were collected. Here, it was found that the lowest TP values were observed in non-evaporated and non-gamma irradiated samples. Because TP contributes to antioxidant activities, these data indicate that evaporation and gamma irradiation may play a role in protecting the antioxidant activities of honey.

The phenolic content of all samples was significantly higher at three months when compared to all other storage durations except in the case of non-gamma irradiated and non-evaporated samples that were stored in clear bottles and kept at room temperature (sample code C). These data indicate that storage conditions and containers may play a vital role in maintaining the phenolic content in honey samples. It is also plausible that in the case of the non-gamma irradiated and non-evaporated samples that were exposed to light and temperature, the antioxidant activity may be compromised from the beginning (three months), such that no change is observed over the duration of storage.

Similarly, the highest content of flavonoids and highest levels of DPPH radical-scavenging activities were also found in evaporated and gamma irradiated Tualang honey samples (Figure 2 and Figure 3). To our knowledge, our study is the first to report that gamma irradiation of Tualang honey samples increases its antioxidant properties. Again, these data indicate that evaporation and gamma irradiation may play a role in protecting the antioxidant activities of honey.

Numerous tests have been developed for measuring the antioxidant activity of food and biological samples. However, there is no universal method that can measure the antioxidant activity of all types of samples accurately. Clearly, matching the radical source and system characteristics to the appropriate antioxidant reaction mechanism is critical in assessing antioxidant activity assay methods [29]. In this study, the DPPH free radical-scavenging assay was used to evaluate the antioxidant activity of the Tualang honey samples. DPPH^·^ is a stable organic nitrogen radical that is commercially available. Assaying scavenging of the DPPH radical is widely used to evaluate antioxidant activity in biological samples as well as in honey samples [2,12,14,30,31,32]. The percentage inhibition of DPPH scavenging over the duration of the assay time reflects the antioxidant activity of the honey assessed. The assay time varies from 10–20 min to approximately 6 h.

The Pearson’s correlation matrix (Table 2) showed that the TP and flavonoid content and DPPH radical-scavenging activities are parameters that can indicate the antioxidant properties of honey. Similarly, a significant linear correlation between the TF and TP content was reported in *Gelam* and *Nenas* honey (r = 0.939) [33]. Socha *et al.* [34] also reported a significant linear correlation (r = 0.83) between the total phenolic content and total flavonoid content in herb honeys. Other studies have also reported good correlations between the antioxidant capacities and phenolic and flavonoid contents, thus indicating that phenolics and flavonoids are some of the major constituents responsible for the antioxidant activity of honey [16,33,34,35,36,37,38].

This is the first study to investigate the effects of temperature, evaporation, type of container (dark and clear bottles) and gamma irradiation on the TP and flavonoid content and DPPH radical-scavenging activities of Tualang honey samples maintained for less than one year in different storage conditions. This study has shown that out of all conditions, gamma irradiation has the most notable potential for changing honey properties, as all gamma irradiated Tualang honey samples showed significantly higher TP and flavonoid content values and free radical-scavenging activities when compared to non-gamma irradiated honey samples. Therefore, processed honey samples should be gamma irradiated in order to maintain antioxidant activities.

Recently, Hussein *et al.* [33] also reported higher antioxidant properties, polyphenol and flavonoid content in gamma irradiated Gelam and Nenas honey samples when compared to non-gamma irradiated honey samples. The effect of gamma irradiation is also important in food samples other than honey. Song *et al.* [39] reported that the antioxidant activity of gamma irradiated carrot juice was higher than that of non-gamma irradiated juice, while Stajner *et al.* [40] found that gamma irradiated soya had a higher antioxidant capacity than non-gamma irradiated soya. Jo *et al.* [41] reported that green tea extracts exposed to gamma irradiation (10 and 20 kGy) showed increased antioxidant properties. Khattak *et al.* [42] reported that gamma gamma irradiation enhanced the free radical-scavenging activity in *Nigella sativa* seeds. It is postulated that the increase in antioxidant activities following gamma irradiation may be due to the degradation of some high-molecular-weight constituents, and changing the solubility of these constituents in test solvents may give rise to the production of additional phenolic compounds [42]. Besides giving a direct effect, gamma irradiation may also give indirect effects where radiolysis of water results in the production of radicals such as hydrated electrons, hydroxyl radicals and hydrogen atoms. These radicals may lead to breakage in the glycosidic bonds in some of the honey’s constituents resulting in the formation of new compounds. The increase in phenolic compounds in gamma-irradiated honeys could be attributed to the release of phenolic compounds from glycosidic components present in honey and the degradation of the larger phenolic compounds into smaller ones by gamma irradiation [43].

## 4. Experimental

### 4.1. Samples

Tualang honey (AgroMas®) was supplied by FAMA (Federal Agricultural Marketing Authority, Malaysia. Various authorized honey collectors collected the honey from Tualang trees growing in the Kedah Rain Forest in January, 2010. The honey sample was filtered using a fine strainer to keep the wax particles out before being heated at 40 °C (the same temperature inside a hive on a hot day) to cause evaporation. This will achieve a 20% water content. The honey samples were then gamma irradiated using a cobalt-60 irradiator at 25 kGy at the Malaysian Nuclear Agency in Selangor, Malaysia.

Samples were assayed at three different time points: The first test was conducted in March 2010 (three months) which was the baseline test of this experiment while the second test was in June 2010 (six months) and the final third test was conducted in December 2010 (12 months) at Pharmacology Laboratory, School of Medical Sciences, University Science Malaysia, Kelantan, Malaysia. The data obtained from the second and third tests were compared with the first test. Other sample conditions and treatments are shown in Table 1.

### 4.2. Chemicals and Reagents

Sodium carbonate, aluminum chloride, sodium nitrite, sodium hydroxide, methanol and ferrous sulphate (FeSO_4_·7H_2_O) were purchased from Merck (Darmstadt, Germany). Gallic acid, chatechin, 1,1-diphenyl-2-picrylhydrazyl radical (DPPH), 2,4,6-tris(2-pyridyl)-1,3,5-triazine (TPTZ) and Folin-Ciocalteu’s reagent were purchased from Sigma-Aldrich (St. Louis, MO USA). All chemicals used in this study were of analytical grade.

### 4.3. Determination of Total Polyphenols

Phenolic compounds were estimated using a modified Folin-Ciocalteu method [44]. Briefly, diluted honey sample (0.2 g/mL, 1 mL) was mixed with Folin-Ciocalteu’s reagent (1 mL). After 3 min, 10% sodium carbonate solution (1 mL) was added to the mixture, and the volume adjusted to with distilled water (10 mL). The reaction was incubated in the dark for 90 min, and the absorbance was then read at 725 nm using a T 80 UV/VIS spectrophotometer (ChromoTek GmbH, Germany). Gallic acid was used to generate the standard curve (20, 40, 60, 80 and 100 µg/mL, r^2^ = 0.996). Samples were analyzed in triplicate. The results are expressed as mean ± standard deviation and are presented in milligrams of gallic acid equivalents (GAEs) per kg honey.

### 4.4. Determination of Total Flavonoids

The total flavonoid (TF) content of honey was determined according to the colorimetric assay developed by Zhishen *et al.* [45]. Briefly, diluted honey (0.2 g/mL, 1 mL) was mixed with distilled water (4 mL). To start, sodium nitrite (NaNO_2_, 5% w/v, 0.3 mL) was added to the honey. Following 5 min of incubation, aluminum chloride (AlCl_3_, 10% w/v, 0.3 mL) was added. Six minutes later, 1 M sodium hydroxide (NaOH, 2 mL) was added. The volume was then immediately adjusted to 10 mL using distilled water (2.4 mL). The mixture was then shaken vigorously, and the absorbance was read at 510 nm. A calibration curve was prepared using standard solutions of catechin (20, 40, 60, 80 and 100 µg/mL, r^2^ = 0.996). The results are expressed as mg catechin equivalents (CEQ) per kg of honey.

### 4.5. DPPH Free Radical-Scavenging Activity

The antioxidant activity of Tualang honey was studied by evaluating the free radical-scavenging effects on the 1,1-diphenyl-2-picrylhydrazyl (DPPH) radical. The assay was based on the method proposed by Ferreira *et al.* [46]. One milliliter of properly diluted honey solution (0.2 g/mL) was mixed with methanolic solution containing DPPH radicals (0.024 mg/mL, 2.7 mL). The mixture was shaken vigorously and left to stand for 60 min in the dark (until stable absorption values were obtained). The reduction of the DPPH radical was determined by measuring the absorbance at 517 nm [47]. The radical-scavenging activity (RSA) was calculated as a percentage of DPPH discoloration using the equation % RSA = [(A_DPPH_ − A_S_) / A_DPPH_] × 100, where AS is the absorbance of the solution following addition of different concentrations of honey and A_DPPH_ is the absorbance of the DPPH solution.

### 4.6. Statistical Analyses

Data were analyzed using SPSS (Statistical Packages for Social Science) 12.0 for Windows (SPSS Inc., Chicago, IL) and Microsoft Office Excel 2003. The statistical differences represented by small letters in all figures were obtained using a one-way analysis of variance (ANOVA) followed by Tukey’s honestly significant difference post hoc tests, with α = 0.05. Correlations were determined using a regression curve fit model (Table 2), where test time points were independent variables and different Tualang honey samples were considered as dependent variables. The assays were conducted in triplicate, and the results are expressed as mean ± standard deviation (SD).

## 5. Conclusions

Our study clearly demonstrates that over twelve months, different storage conditions had little effect on the polyphenol and flavonoid contents and DPPH radical-scavenging activities in Tualang honey samples. However, gamma irradiation had the potential to maintain these properties successfully. The most notable finding of our study was that evaporation, the type of container (dark or clear bottles) and temperature showed minor effects on the phenolic and flavonoid contents and DPPH radical-scavenging activities, while gamma irradiation played a major role in influencing the antioxidant properties of honey. Longer storage durations were associated with gradual decreases in the polyphenol and flavonoid contents and radical-scavenging activities of honey samples stored in different ways. Further research is now required to reveal the mechanism underlying the increase in polyphenol content and radical-scavenging activities of gamma irradiated Tualang honey samples.
